# Short Inflammatory Bowel Disease Questionnaire: translation and validation to the Portuguese language

**DOI:** 10.1186/s12955-021-01698-9

**Published:** 2021-02-18

**Authors:** Joana Roseira, Helena T. Sousa, Ana Marreiros, Luís F. Contente, Fernando Magro

**Affiliations:** 1 Gastroenterology Department, Algarve University Hospital Center, Portugal, Estr. Poço Seco, 8500-338 Portimão, Portugal; 2 ABC, Algarve Biomedical Center, University of the Algarve, Portugal, Estr. da Penha, 8005-139 Faro, Portugal; 3 Department of Pharmacology and Therapeutics, Faculty of Medicine of Porto, Rua Plácido Costa, 4200-450 Porto, Portugal

**Keywords:** HRQoL, Inflammatory bowel disease, Patient reported outcome measure, Translation, Validation

## Abstract

**Background:**

The Short Inflammatory Bowel Disease Questionnaire (SIBDQ) is a widely used instrument to assess Health-related Quality of Life (HRQoL) among inflammatory bowel disease (IBD) patients. Our aim was to translate and adapt the SIBDQ so that it could be adequately used in Portugal.

**Methods:**

This is a prospective design cohort study undertaken at a tertiary hospital. This study took place simultaneously with the first part of the *SexIDI* study, a study aiming to assess the impact of IBD on patients’ sexual QoL. The original SIBDQ was translated by two independent translators and adapted by an IBD expert panel following the opinions of a convenient sample of 5 IBD patients. Afterwards, IBD patients from the outpatient clinic were consecutively invited to fill the Portuguese version of the questionnaire (SIBDQ-PT) at three different timepoints (0, 2, 4 weeks). Ninety-two patients completed the SIBDQ-PT at baseline, whereas 33 did so after 2 and 4 weeks (approximately). Statistical analysis was performed using SPSS version 25, and the following aspects were analysed: reliability (through internal consistency, test–retest and intraclass correlation), validity (through exploratory factor analysis [EFA], and Pearson correlation coefficient for linear correlations), score distribution, and responsiveness analysis (through t-student tests).

**Results:**

Overall, SIBDQ-PT was shown to have a high internal consistency (Cronbach's α = 0.80) and a high test–retest reliability (0.80 [CI 0.74–0.86] and 0.69 [CI 0.50–0.82]). EFA detected four dimensions—bowel, social, emotional and systemic. As expected, an overall SIBDQ-PT score was positively correlated with sexual satisfaction (r = 0.27; *p* < 0.05) and negatively correlated with depression (r = − 0.63; *p* < 0.01). Moreover, SIBDQ-PT was found to have an adequate score distribution, and to be responsive, as there was a significant subscore change for patients who reported an “overall worsening in general well-being” (0.93 ± 0.13 decrease; *p* < 0.01).

**Conclusions:**

The Portuguese version of the SIBDQ hereby presented is a reliable, valid and responsive instrument that can be used to measure HRQoL among Portuguese IBD patients.

## Background

Inflammatory bowel diseases (IBD) are a group of chronic diseases characterised by an early onset and by a relapse and remission pattern. Prevalence has increased over time, mostly due to the low mortality rates associated with these conditions [1]. Prevalence estimates in Portugal are, currently, among the highest in Europe [2, 3]. The unpredictable onset of disease flares, the associated symptoms and the effects of treatment regimens strongly impact patients’ health-related quality of life (HRQoL) and, therefore, this is currently acknowledged as an important patient-reported outcome in IBD [4].

Chen et al*.* recently conducted a critical review of HRQoL instruments specifically designed for IBD patients [5]. Among the many assessment tools, the IBD Questionnaire, developed by Guyatt et al*.*, was considered to be among the most suitable, valid and reliable. Irvine and colleagues validated a short, self-administered version of the IBD Questionnaire, which was also found to be valid and reliable in assessing the HRQoL of Crohn’s disease (CD) and ulcerative colitis (UC) patients’ [5–7]. This Short Inflammatory Bowel Disease Questionnaire (SIBDQ) version became widely known and is currently used worldwide both in clinical practice and clinical research. The SIBDQ comprises a total of 10 questions grouped into four different dimensions: social, bowel, emotional, and systemic [6, 7]. Each question is scored by a 7-point Likert scale, ranging from 1 (a severe problem) to 7 (not a problem at all), giving an absolute SIBDQ score ranging from 10 (poor HRQoL) to 70 (optimal HRQoL). A SIBDQ score below 50 was considered as poor QoL. There are no validated cut-offs for the different dimensions’ scores. Hence, higher scores indicate a better HRQoL concerning that specific domain.

Although patient-reported outcomes are highly valuable for better patient care, patient responses to HRQoL instruments can be impacted by underlying cultural trends [8]. Therefore, the direct translation of HRQoL questionnaires does not guarantee their adequacy and utility in countries others than those in which the questionnaire was designed and initially validated. As stressed, the SIBDQ is among the best-characterised tools to access IBD HRQoL. Since its development, it has been increasingly used in observational studies, clinical trial and clinical practice settings. However, a Portuguese translation and validation of the SIBDQ was yet to be performed.

This article describes the adaptation and validation of the Portuguese version of the SIBDQ (SIBDQ-PT). Our aim was to adapt an international instrument so that it could effectively and adequately evaluate HRQoL among IBD patients in Portugal.

## Materials and methods

### SIBDQ translation and adaptation

The original version was independently translated forward (into Portuguese) by two bilingual translators (J.R., H.T.S.). The translations were reconciled to address discrepancies between the original forward independent translations. A single literal back translation (to English) was then performed by a Portuguese gastroenterology resident (J.R) who is a native language speaker with certified English proficiency. The original version and the back-translated version of the SIBDQ were compared and harmonised to ensure conceptual equivalence between the source and target language versions (J.R., H.T.S.). Moreover, a convenient sample of 5 Portuguese IBD patients evaluated the questionnaire in terms of interpretation/ease of comprehension and made their suggestions for improvement. Finally, a panel discussion with the project manager (J.R.) and bilingual experts in IBD (H.T.S. and F.M.) took place to check the final translation and to reformulate some items according to the cognitive debriefing of the 5 patients.

### Validation of the SIBDQ-PT

This study took place simultaneously with the first part of the SexIDI study, a cross-sectional study aiming to assess the impact of IBD on patients’ sexual QoL [9]. Patients attending the IBD outpatient clinic of Portimão Hospital with 18 to 65 years of age and an IBD diagnosis for at least 2 years, were considered eligible. The Portimão Hospital is part of the Algarve Hospital University Center that includes the “Portimão unit” and the “Faro unit”. This is a general, tertiary care, public Hospital center; the Portimão unit provides care to approximately 500 gastroenterology outpatients monthly (according to January to March 2019 hospital statistics). Two clinicians (J.R. and H.T.S.) conducted eligibility assessments and invited patients to participate in the study. One hundred, consecutive patients were invited to participate between July and December 2016. Patients willing and able to participate signed an informed consent form prior to inclusion.

At baseline, patients were given a multimodal form including not only the SIBDQ-PT, but also questions regarding socio-demographic variables (age, sex, nationality, educational level, professional status, relationship status), clinical variables (height and weight, type of IBD and disease duration), and three already validated scores: (1) the short version of the Social Desirability Scale (SDS-SF) [10], which measures the tendency to engage in socially desirable responding; (2) the Sexual Quality of life questionnaire Male/Female (SQol M/F) [11], which focuses on patients’ feelings about their sexual life; and (3) the Nine-item Patient Health Questionnaire (PHQ-9) [12], which is a depression spectrum disorder symptom scale. The SIBDQ-PT was later applied in two other timepoints, approximately 2 and 4 weeks after baseline, and at the last timepoint patients were also questioned about whether they felt an “overall worsening in their general well-being”. In all cases the questionnaires were anonymous and self-administered.

### Statistical analysis

#### Acceptability

The acceptability of the SIBDQ-PT was determined according to the degree to which the questionnaires were filled. Whenever more than 20% of answers were missing from a questionnaire, that questionnaire was excluded from the analysis. The average time to complete the questionnaire was auto-assessed for the initial 5-patient sample.

#### Item reduction and data structure

Exploratory Factor Analysis (EFA) with Principal Components (PC) Extraction method was used to detect the underlying structure of the 10 items included in the SIBDQ-PT, and to confirm the multidimensionality of the questionnaire previously determined by Irvine et al. [6, 7] Kaiser–Meyer–Olkin measure (KMO) of adequacy and Bartlett's test of sphericity were directed to check sample adequacy for carrying out an EFA [13]. The number of factors was set using the Kaiser rule (latent roots equal or greater than one) computed from the correlation matrix of all original items. The determinant of the matrix was used to test for multicollinearity or singularity (the determinant should be greater than 0.00001). A Varimax orthogonal rotation was performed. The rotation has the effect of optimizing the factor structure and equalizing relative importance of factors. An orthogonal rotation was preferred to maintain the assumption of the orthogonality of the dimensions, and to allow the results to have the greatest possible independence of the axis formed by the new linear combinations of the original variables. The number of components was set by examining the plot of eigenvalues [14]. The final components were supported according to the total variance explained and the solution interpretability. Finally, the communalities reflected the common variance of the data structure.

#### Reliability

Reliability (the extent to which scores tend to remain the same for a patient under the same conditions) was measured using all items from the health-related patient-reported outcome (HrPRO) (internal consistency), and over time (test–retest) [15].

Internal consistency was assessed using Cronbach’s α (employs covariances among the items) and Cronbach’s α based on standardized items (employs the correlations among items). Cronbach’s α 0.7–0.9 corresponds to an adequate homogeneity of the items [16].

Test–retest reliability was assessed using the intraclass correlation coefficient (ICC) [15] between the results obtained in the two initial timepoints. An interval of two weeks was considered long enough to prevent recall-bias and short enough to ensure similar disease status. ICC was calculated on the basis of an absolute agreement and the 95% confidence intervals (CIs) were verified [17]. An ICC 0.60–0.75 is classified as “good” [18]. The standard error of measurement was calculated as *standard deviation x* $\sqrt {(1} - ICC)$*.*

#### Validity

Validity refers to the degree to which a HrPRO measures the variable it is meant to. Structural validity was assessed through the above mentioned EFA and construct validity was evaluated using the hypothesis testing method [15, 19]. Pearson’s correlations between SIBDQ-PT/SQoL and SIBDQ-PT/PHQ-9 administered at baseline were calculated. The following hypotheses were a priori formulated: (1) the SIBDQ-PT overall score positively correlates with sexual QoL (SQoL); (2) the SIBDQ overall score negatively correlates with depression symptoms (PHQ-9). A Pearson’s correlation coefficient 0.3–0.5 is classified as “fair”, between 0.5 and 0.8 as “moderately strong”, and greater than 0.8 as “very strong” [20].

#### Score distribution

Score distribution or measurement bias refers to the distribution of the scores in the entire sample, taking into account deviations such as floor and ceiling effects [15, 19]. These effects are considered to be offending if more than 15% of the patients have the minimum or the maximum score.

#### Responsiveness

Responsiveness refers to the sensitivity to change of the questionnaire [15]. The Student’s t-test for paired measurements was used to compare the mean scores for SIBDQ-PT individual items between baseline and the third timepoint for patients who referred an “overall worsening in general well-being”. Individual items scores were also compared for those who did not perceive disease worsening.

Observed data was described as mean ± standard deviation for continuous variables and absolute and relative frequencies for categorical variables. All statistical analyses were performed using SPSS version 25.0 (IBM Corp., Armonk, New York, USA), and level of significance of 5% was established.

## Results

### SIBDQ translation and cultural adaptation

Translation and cultural adaptation of the SIBDQ followed a multiphase process: forward translation, reconciliation, back translation, review and harmonization, comprehensibility assessment by an IBD sample of 5 patients, a panel consensus meeting to review the cognitive debriefing results from the 5-patient sample, and finally proofread the translated scale. Minor discrepancies during the forward and back translation phases included the use of prepositions and sentence structure. Additionally, one unresolvable problem was identified and clarified after the final panel discussion: for items 6 and 7, the first Likert-scale response option “a major problem”, could be translated to “problema major” or “problema severo” that can be used synonymously in Portuguese. The first solution was chosen for its similarity to the original English scale. Finally, the expert IBD panel reviewed the SIBDQ-PT translation and the questions raised by the initial sample of 5 patients and made three main adaptations to the Portuguese version of the SIBDQ:

1. Item 3—“how much difficulty” was changed to “quantify the difficulty” (“quantifique a dificuldade” in Portuguese), in order to obtain a more grammatically appropriate translation.
2. Item 7—The interpretation of the expression “weight you would like to be” raised some doubts, as all five patients that evaluated the questionnaire reported they would like to weigh less than what they acknowledge would be their ideal weight. Therefore, the term was changed to “ideal weight” (“peso ideal” in Portuguese), meaning the ideal weight before the modifications imposed by the disease.
3. Item 8—This item addresses the frequency with which patients have been feeling relaxed, and the 7-point Likert scale in the original version was organized from 1 (“none of the time”) to 7 (“all of the time”). However, throughout the rest of the questionnaire (namely items 1, 2, 4, 5, 9 and 10), the 7-point Likert scale is organized from 1 (“all of the time”) to 7 (“none of the time”). Therefore, 3 of the 5 patients from the initial sample reported an initial impulse to answer as if options were also sequenced from “all of the time” to “none of the time”. Accordingly, the answers to question number eight in the SIBDQ-PT were reorganised to follow the same sequence as the answers to the other questions.

### Sociodemographic, clinical data and score results

At baseline, 92 of the 100 patients who were invited accepted to participate in this study and fulfilled the multimodal questionnaire at first timepoint (i.e. baseline), whereas only 33 (35.87%) were available to do so in the second and third timepoints. The sociodemographic and clinical characteristics of the study population are shown in Table 1. Patients were mostly female (57.61%), tended to be middle-aged (40–49 years, 31.52%), employed (76.09%), married (45.65%), and had mostly completed high-school (52.17%). No major differences were found between age ranges [(18–39 years old; n = 42, 45.65%); (40–65 years old; n = 50, 54.35%)] and gender [(female; n = 53, 57.61%); (male; n = 39, 42.39%)]. Age and gender were also comparable in the sample of participants who completed the study questionnaires in the subsequent timepoints (Table 1). On the other hand, the sample comprised patients with somewhat different educational levels, working status and relationships. Overall, 38 (41.30%) patients had CD and 54 (58.70%) had UC, and the median disease duration was 5 years (interquartile range 15.5). The mean ± standard-deviation of the SIBDQ-PT score obtained at baseline was 52.80 ± 9.45, with a median score of 54.00. The median scores for the SDS-FS, SQoL and PHQ-9 scales, also fulfilled at baseline, were analysed separately for patients that scored below or above the median SIBDQ-PT (Table 2). The mean ± standard-deviation of the SIBDQ-PT score obtained at the second and third timepoints was 53.79 ± 7.96 and 53.82 ± 7.82, respectively.

**Table 1:** Sociodemographic and clinical characteristics

Variables	Baseline sample	Second and third timepoints sample
	(n = 92)	(n = 33)
Age, n (%)		
[18–20]	3 (3.26)	2 (6.06)
[21–29]	21 (22.83)	6 (18.18)
[30–39]	18 (19.57)	11 (33.33)
[40–49]	29 (31.52)	9 (27.27)
[50–59]	15 (16.30)	4 (12.12)
> 60	6 (6.52)	1 (3.03)
Sex, n (%)		
Female	53 (57.61)	17 (51.52)
Male	39 (42.39)	16 (48.48)
Anthropometric data, mean ± SD		
Weight	68.97 ± 14.23	69.30 ± 13.41
Height	167.69 ± 9.99	167.97 ± 9.81
Educational status, n (%)		
Primary schooling	15 (16.30)	4 (12.12)
High schooling	48 (52.17)	18 (54.55)
Graduation degree	27 (29.35)	10 (30.30)
Master’s degree	2 (2.17)	1 (3.03)
Current professional status, n (%)		
Employed	70 (76.09)	21 (63.64)
Unemployed, looking for a job	11 (11.96)	7 (21.21)
Unemployed, not looking for a job	2 (2.17)	2 (6.06)
Retired	7 (7.61)	2 (6.06)
Temporary disability	2 (2.17)	1 (3.03)
Marital status, n (%)		
Married	42 (45.65)	14 (42.42)
Divorced	14 (15.30)	4 (12.12)
Single/dating	36 (39.13)	15 (45.45)
Type of IBD, n (%)		
Crohn’s disease	38 (41.30)	14 (42.42)
Ulcerative colitis	54 (58.70)	19 (57.58)
Duration of the disease, median (range)	5.00 (2–40)	5.00 (3–40)

*SD* standard deviation

**Table 2:** Median baseline scores and standard deviation for the SDS-FS, SQoL and PHQ-9 according to the median SIBDQ-PT

	Social desirability (SDS-FS)	Sexual QoL (SQoL)	Depression screening (PHQ-9)
SIBDQ-PT < 54	10.00 ± 2.09	57.57 ± 21.48	6.00 ± 4.88
SIBDQ-PT ≥ 54	10.00 ± 1.64	83.33 ± 17.23	2.00 ± 3.06
Difference	0.00	25.76	4.00
*p* value	0.92	0.01	0.00

*SIBDQ-PT* Portuguese version of the Short Inflammatory Bowel Disease Questionnaire, *SDS-FS* short version of the Social Desirability Scale, *PHQ-9* the Nine-item Patient Health Questionnaire

### SIBDQ-PT validation

#### Acceptability

The 5 patients who initially evaluated the SIBDQ-PT reported to have spent an average of 3 min to complete the questionnaire.

In terms of the multimodal questionnaire administered at baseline, all patients completed the SIBDQ-PT and the PHQ-9 scores. However, 3.26% of patients did not complete the SDS-SF score (two questionnaires had over 80% of missing values and were, therefore, excluded from the analysis), and 14.13% of patients did not complete the SQoL questionnaire (but no questionnaire had over 80% of missing values, and therefore all completed questionnaires were included in the analysis). As for the second and third timepoints, all patients completed the SIBDQ-PT questionnaire.

#### Item reduction and data structure

The correlation matrix was used to verify the pattern to be observed between the 10 items that composed the SIBDQ-PT: the correlation coefficients were no greater than 0.9 and the determinant of the correlation matrix was 0.02 which is greater than the necessary value of 0.00001. Thus, singularity and multicollinearity of the data were excluded, and there was no need to consider eliminating any item of the questionnaire.

Eigenvalues above one allowed to reduce the initial data dimension to three factors, explaining over 60% of questionnaire variance. However, due to the fourth eigenvalue proximity to one (0.933), and because it translated into an accumulated contribution of variance of 74.49%, a four-factor solution was considered. The Pearson correlation coefficients between the items and the factors are shown in Table 3. Factor one is expressed by items 4, 6, 9 and 10; factor two is expressed by items 2, 3 and 5; factor three is expressed by items 1 and 8; factor four in expressed by item 7. Keeping the terminology used in the original questionnaire, the four factors detected in the SIBDQ-PT were named as the bowel dimension, the social dimension, the emotional dimension and the systemic dimension.

**Table 3:**
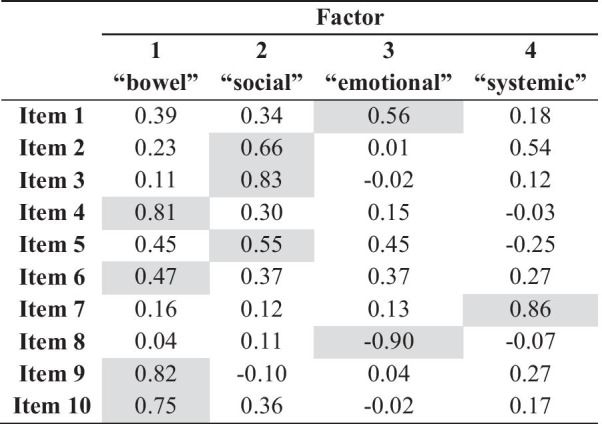
Rotated component matrix

Extraction method: principal component analysis;

Rotation method: Varimax with Kaiser Normalization; rotation converged in 7 iterations

The sample adequacy was supported by a KMO > 0.79 (meritorious) and a Bartlett's Test of Sphericity *p* value < 0.01. The communalities values of each item ranged between 0.58 (item 6) and 0.84 (item 8).

#### Reliability

The SIBDQ-PT Cronbach’s α coefficient was 0.80, which indicates an adequate homogeneity of the items. Moreover, the test–retest reliability was high, as indicated by the ICC between the baseline and the second SIBDQ-PT administration: 0.69 (95% CI: 0.50–0.82). Considering the SIBDQ-PT retest and reliability results, the estimated standard error was 4.43.

#### Validity

We have a priori hypothesised that the SIBDQ-PT should be positively correlated to sexual QoL and negatively correlated with the incidence of depressive symptomatology. These hypotheses held true, as an overall SIBDQ-PT score was found to be positively correlated with sexual satisfaction (r = 0.27; *p* < 0.05) and negatively correlated with depression symptoms (r = − 0.63; *p* < 0.01).

#### Score distribution

The floor and ceiling effects detected in the test and retest (second timepoint) were considered to be irrelevant. At baseline, only two and one patients had an overall score at the lower and upper end of the scale, respectively. At the retest, one patient had an overall score at the lower end of the scale, and one other patient had a score at the upper end of the scale.

#### Responsiveness

Out of the 33 patients who completed the SIBDQ-PT on the third timepoint (30 to 43 days after baseline), 9 patients reported an “overall worsening in their general well-being”: their individual items mean scores (range 1–7) were statistically significantly lower than at baseline: 5.63 *vs.* 4.70 (0.93 ± 0.13 decrease; *p* < 0.01). For those who did not perceive disease worsening (n = 24), individual scores remained comparable (0.01 ± 0.03 decrease: *p* = 0.08).

## Discussion

The importance of patient-reported outcomes, namely in what concerns HRQoL, is increasingly acknowledged as a key aspect in the management of IBD patients. HRQoL is a multidimensional concept that can be viewed as a latent construct encompassing physical, social and psychological aspects of well-being and role functioning [21]. But despite the far-reaching nature of this definition, the importance of using culturally-validated instruments in local language is often ignored. Consequently, the quality of psychometric data falls behind that of the somatic data. With this study we aimed to translate and validate the widely used SIBDQ so that it could be adequately used in Portugal.

Eight translations of the original IBDQ have been validated in Europe, as well as two translations of the SIBDQ [22–31]. No IBDQ nor SIBDQ translations have, to the best of our knowledge, been validated in Portugal. Some reviews do refer to a Portuguese validation of the IBDQ, but this validation was made in the Brazilian context [32].

After independent forward and backward translations, context-validation by an IBD cohort, and an expert panel meeting, we have translated the SIBDQ to Portuguese (SIBDQ-PT). Also, as a result of this study the SIBDQ-PT was found to be structurally valid, reliable, convergent with sexual satisfaction and depression, to have an appropriate score distribution and to be responsive to change. In order to assess the psychometric data of the SIBDQ-PT, we have applied the questionnaire at three different timepoints separated by a two-week interval. The baseline questionnaire was self-administered to a panel of 92 IBD outpatients accounting for 92% of invited individuals. This panel size was considered to be appropriate, having into account that the original SIBDQ comprises a total of 10 items and the minimum patient-to-item ratio recommended to run a factor analysis is 7:1 [19]. Regarding the second and third timepoints, and despite the fact that all 92 patients were invited to participate, only 33 were available to do so. Still, the sample size was appropriate to check the parameters that depended on the repetition of the questionnaire (re-test and responsiveness analysis).

The SIBDQ-PT construct validity was demonstrated through the confirmation of the a priori formulated hypotheses: an overall SIBDQ-PT score was positively correlated with sexual QoL (SQoL) and negatively correlated with depression symptoms (PHQ-9). The study conducted in the context of the British translation [28] unveiled the presence of a strong correlation between the HRQoL, measured by the SIBDQ, and physician-evaluated disease activity indexes namely the Simple Clinical Colitis Activity index (SCCAI) and the Seo index: r = 0.83 (*p* < 0.01) for the SCCAI and r = 0.61 (*p* < 0.01) for the Seo index. However, the original American SIBDQ [6, 7] acknowledges the existence of psychosocial dimensions other than the “bowel dimension”. Moreover, IBD-focused studies have shown that the patient self-perception of the illness is a better predictor of QoL, than IBD activity [33–35]. Thus, this study aimed to be fully based on patient-reports*.* We have, therefore, investigated the presence of correlations between HRQoL and other patient-reported concerns/symptoms like sexual QoL and depression, and identified a fair and a strong correlation, respectively. Sexual QoL may be a very different construct which is related to IBD-specific HRQoL but only distally, accounting for the fair correlation. Depression on the other hand, seems to be a measure better fitting this “patient perception of the illness” umbrella to test convergent validity of the SIBDQ. Construct validity was validated by the hypothesis testing method [19] using only patients self-rated measures.

The results of the EFA analysis confirmed that the SIBDQ-PT measures different components of QoL. Similarly to what is observed in the original version [6, 7], four dimensions—bowel (expressed by items 4, 6, 9 and 10), social (expressed by items 2, 3 and 5), emotional (expressed by items 1 and 8) and systemic (expressed by item 7)—were perceived in the Portuguese version of the SIBDQ. However, if we compare the original version and the Portuguese version (see Additional file 1), we notice that these dimensions were not expressed exactly by the same items. Item 10, for example, refers to the time during which the patient “felt angry as a result of his/her bowel problem”. In the original American SIBDQ this item is included in the emotional dimension, whether in the Portuguese version it is included in the bowel dimension. This question refers to “anger” related to the “bowel problem”, thus it adequately expresses both dimensions depending on the tone we put on the emotion and on the bowel condition. This is highly influenced by patients’ interpretations in different contexts, according to their language and population structure, and further reinforces the importance of using language-validated instruments. Items one and 5, referring to “exhaustion” and “demotivation”, also express different dimensions comparing both SIBDQ (systemic and emotional) and SIBDQ-PT (emotional and social), and a similar explanation applies. As so, we believe our four-dimension solution is not only supported by the total variance explained (over 70%), but also by its interpretability. As for the dimensions’ individual scores, alike the original SIBDQ there are no validated cut-offs. Hence, higher scores indicate a better HRQoL concerning that specific domain.

SIBDQ-PT was shown to have high internal consistency and test–retest reliability. The assessment of the score distribution included the evaluation of SIBDQ-PT total scores and the assessment of floor/ceiling effects, which were absent both at baseline and on the re-test. Sensitivity to change was stressed by the responsiveness analysis for patients who self-reported an overall worsening in their condition.

This study has several strengths that should be highlighted. Since the only criteria for participation in this study was age 18–65 years and a diagnosis for at least two years, the study population was community-based and, therefore, likely representative of all stages of the disease. As people tend to differ in their tendency to engage in socially desirable responding (SDR), which is a concern when analysing self-administered questionnaires, a SDR questionnaire—the SDS-FS [10]—was administered in parallel with the SIBDQ-PT. Additionally, and despite the absence of heterogeneity in terms of age and gender distribution, the sample included patients with different educational levels and working/relationship status, and was therefore considered to be adequate for a cultural adaptation and validation [5]. To our knowledge, this is the first study evaluating the score distribution and measurement bias of SIBDQ—neither the original version developers nor the authors of the British and German translations assessed this aspect [28, 29]. Another important strength is the fact that the questionnaire was self-administered, ensuring patients were given the same instructions and the instrument was fulfilled under the same conditions. Data collection in other translation validations is performed over the phone and with different interviewers, which may influence the results. Nevertheless, this study also has limitations that should be noted. The study was conducted at a single centre, the sample is modest and recruited by convenience. Still this sample is assumed to be representative of the Portuguese population for the purposes of this translation, allowing adequate and complete psychometric assessment.

## Conclusions

Assessing HRQoL in an objective and reliable way is now considered to be a prerequisite of well-designed IBD trials. Therefore, careful validation of the SIBDQ in different languages is an essential step to allow the comparison of data across different countries in multinational trials. As a result of this study, we now have a validated Portuguese version of the SIBDQ available for use in both clinical practice and clinical trials (Additional file 1).

## Supplementary Information

**Additional file 1.** Portuguese version of the Short Inflammatory Bowel Disease Questionnaire & Original American version.

## Data Availability

The authors confirm that the data supporting the findings of this study are available within the article and its additional materials**.**

## Abbreviations

SIBDQ: The Short Inflammatory Bowel Disease Questionnaire
HRQoL: Healt-related Quality of Life
IBD: Inflammatory bowel disease
SIBDQ-PT: Portuguese version of the Short Inflammatory Bowel Disease Questionnaire
PCA: Principal component analysis
KMO: Kaiser–Meyer–Olkin measure
HrPRO: Health-related patient-reported outcomes
CD: Crohn’s disease
UC: Ulcerative colitis
SDS-FS: Short version of the Social Desirability Scale
SQoL M/F: Sexual Quality of life Questionnaire Male/Female
PHQ-9: The nine-item Patient Health Questionnaire
ICC: Intraclass correlation coefficient
SDR: Socially desirable responding
SCCAI: Simple Clinical Colitis activity index
