# Supplementary material for: Short Inflammatory Bowel Disease Questionnaire: translation and validation to the Portuguese language
Source: Health Qual Life Outcomes. 2021 Feb 18;19:59. doi: 10.1186/s12955-021-01698-9 (PMC7891007; doi:10.1186/s12955-021-01698-9)
Supplement: Supplementary file 1 — Additional file 1. Portuguese version of the Short Inflammatory Bowel Disease Questionnaire & Original American version. [file 12955_2021_1698_MOESM1_ESM.docx]

Additional file 1

**SIBDQ-PT**

**1. Nas últimas 2 semanas, com que frequência é que a sensação de cansaço ou exaustão foi um problema para si? *[emotional]***

Sempre [ ]; maior parte do tempo [ ]; uma boa parte do tempo [ ]; algumas vezes [ ]; poucas vezes [ ]; raramente [ ]; nunca [ ]

**2. Nas últimas 2 semanas, com que frequência teve de adiar ou cancelar um evento social devido à sua doença intestinal? *[social]***

Sempre [ ]; maior parte do tempo [ ]; uma boa parte do tempo [ ]; algumas vezes [ ]; poucas vezes [ ]; raramente [ ]; nunca [ ]

**3. Nas últimas 2 semanas, quantifique a dificuldade que teve em realizar atividades de lazer ou desportivas de que gosta, devido à sua doença intestinal. *[social]***

Extrema dificuldade, impossível realizar as atividades [ ]; muita dificuldade [ ]; bastante dificuldade [ ]; alguma dificuldade [ ]; pouca dificuldade [ ]; praticamente nenhuma dificuldade [ ]; nenhuma dificuldade, sem limitações de pratica desportiva e atividades de lazer [ ]

**4. Nas últimas 2 semanas, com que frequência se sentiu incomodado com dor abdominal? *[intestinal]***

Sempre [ ]; maior parte do tempo [ ]; uma boa parte do tempo [ ]; algumas vezes [ ]; poucas vezes [ ]; raramente [ ]; nunca [ ]

**5. Nas últimas 2 semanas, com que frequência se sentiu deprimido ou desmotivado? *[social]***

Sempre [ ]; maior parte do tempo [ ]; uma boa parte do tempo [ ]; algumas vezes [ ]; poucas vezes [ ]; raramente [ ]; nunca [ ]

**6. De uma forma geral, nas últimas 2 semanas, quão problemática foi para si a flatulência? *[intestinal]***

Problema *major* [ ]; grande problema [ ]; problema significativo [ ]; algum incómodo [ ]; pequeno incómodo [ ]; praticamente nenhum incomodo [ ]; não foi problema [ ]

**7. De uma forma geral, nas últimas 2 semanas, quão problemática foi a manutenção do seu peso ideal? *[sistémico]***

Problema *major* [ ]; grande problema [ ]; problema significativo [ ]; algum incómodo [ ]; pequeno incómodo [ ]; praticamente nenhum incomodo [ ]; não foi problema [ ]

**8. Nas últimas 2 semanas, com que frequência se sentiu relaxado? *[emocional]***

Sempre [ ]; maior parte do tempo [ ]; uma boa parte do tempo [ ]; algumas vezes [ ]; poucas vezes [ ]; raramente [ ]; nunca [ ]

**9. Nas últimas 2 semanas, com que frequência se sentiu incomodado com a sensação de ter de ir à casa de banho, mesmo não tendo dejeções? *[intestinal]***

Sempre [ ]; maior parte do tempo [ ]; uma boa parte do tempo [ ]; algumas vezes [ ]; poucas vezes [ ]; raramente [ ]; nunca [ ]

**10. Nas últimas 2 semanas, com que frequência se sentiu irritado como resultado da sua doença intestinal? *[intestinal]***

Sempre [ ]; maior parte do tempo [ ]; uma boa parte do tempo [ ]; algumas vezes [ ]; poucas vezes [ ]; raramente [ ]; nunca [ ]

**SIBDQ – Original American version**

**1. How often as the feeling of fatigue or of being tired and worn out been a problem for you during the last 2 weeks? *[systemic]***

All of the time [ ]; most of the time [ ]; a good bit of the time [ ]; some of the time [ ]; a little of the time [ ]; hardly any of the time [ ]; none of the time [ ]

**2. How often during the last 2 weeks have you had to delay or cancel a social engagement because of your bowel problem? *[social]***

All of the time [ ]; most of the time [ ]; a good bit of the time [ ]; some of the time [ ]; a little of the time [ ]; hardly any of the time [ ]; none of the time [ ]

**3. How much difficulty have you had, as a result of your bowel problems, doing leisure or sports activities you would have liked to have done during the last 2 weeks? *[social]***

A great deal of difficulty, activities made impossible [ ]; a lot of difficulty [ ]; a fair bit of difficulty [ ]; some difficulty [ ]; a little difficulty [ ]; hardly any difficulty [ ]; no difficulty; the bowel problems did not limit sports or leisure activities [ ]

**4. How often during the last 2 weeks have you been troubled by pain in the abdomen? *[bowel]***

All of the time [ ]; most of the time [ ]; a good bit of the time [ ]; some of the time [ ]; a little of the time [ ]; hardly any of the time [ ]; none of the time [ ]

**5. How often during the last 2 weeks have you felt depressed or discouraged? *[emotional]***

All of the time [ ]; most of the time [ ]; a good bit of the time [ ]; some of the time [ ]; a little of the time [ ]; hardly any of the time [ ]; none of the time [ ]

**6. Overall, in the last 2 weeks, how much of a problem have you had with passing large amounts of gas? *[bowel]***

A major problem [ ]; a big problem [ ]; a significant problem [ ]; some trouble [ ]; a little trouble [ ]; hardly any trouble [ ]; no trouble [ ]

**7. Overall, in the last 2 weeks, how much of a problem have you had maintaining or getting to the weight you would like to be? *[systemic]***

A major problem [ ]; a big problem [ ]; a significant problem [ ]; some trouble [ ]; a little trouble [ ]; hardly any trouble [ ]; no trouble [ ]

**8. How often during the last 2 weeks have you felt relaxed and free of tension? *[emotional]***

None of the time [ ]; a little of the time [ ]; some of the time [ ]; a good bit of the time [ ]; most of the time [ ]; almost all of the time [ ]; all of the time [ ]

**9. How much of the time during the last 2 weeks have you been troubled by a feeling of having to go to the bathroom even though your bowels were empty? *[bowel]***

All of the time [ ]; most of the time [ ]; a good bit of the time [ ]; some of the time [ ]; a little of the time [ ]; hardly any of the time [ ]; none of the time [ ]

**10. How much of the time during the last 2 weeks have you felt angry as a result of your bowel problem? *[emotional]***

All of the time [ ]; most of the time [ ]; a good bit of the time [ ]; some of the time [ ]; a little of the time [ ]; hardly any of the time [ ]; none of the time [ ]
